# Detecting Citation Veneer in Guideline-Grounded Clinical and Public Health Large Language Model (LLM) Outputs: A Clinical Evidence Audit Grid Using CENHOV Tags

**DOI:** 10.7759/cureus.107729

**Published:** 2026-04-26

**Authors:** Yuusuke Harada

**Affiliations:** 1 Public Policy, Hosei University, Tokyo, JPN; 2 Humanities and Social Sciences, Hiroshima University, Hiroshima, JPN

**Keywords:** citation veneer, clinical decision support, clinical informatics, hallucination auditing, large language models, patient education, patient safety, public health, quality assurance, retrieval-augmented generation

## Abstract

Large language model (LLM) systems are increasingly used to summarize clinical guidelines, draft patient-facing education, and answer evidence-linked clinical or public health questions. We define citation veneer as an observable audit state in which an output presents citation cues or apparently supportive source information while still containing incorrect, incomplete, or materially unsupported content. We develop and evaluate the Clinical Evidence Audit Grid (CEAG), an analyst-facing monitoring surface that encodes six binary response-quality signals into a fixed-length plain-text audit tag (CENHOV): clinical correctness (C), evidential support (E), numeric concordance (N), hedging (H), stylistic ornamentation (O), and a derived CEAG-V supported-error citation veneer marker (V=1 when E=1 and C=0). CEAG was evaluated on a controlled benchmark (synthetic, n=120 questions per condition) and on a 12-case public check built from PubHealth-style health-claim examples and public medical or public-health sources. The controlled benchmark used scripted response regimes rather than a live LLM/API benchmark; therefore, model identity, sampling temperature, and seed are not applicable to the main controlled analysis. In the synthetic benchmark, the RUSH regime retained high evidential-support rates (93.3%, 95% CI 87.4%-96.6%) while dropping to 73.3% correctness (95% CI 64.8%-80.4%) and producing 25.8% CEAG-V veneer (95% CI 18.8%-34.3%). In the 12-case public check, RUSH produced 66.7% CEAG-V veneer (95% CI 39.1%-86.2%), whereas VERIFY and POSTER produced none; because n=12, these public-case rates are descriptive pilot data only. CEAG is not proposed as a patient-facing display or a replacement for conventional charts. It is a clinical informatics monitoring aid for reviewers who need to detect apparently well-sourced hallucinations, compare response regimes, and separate stylistic change from substantive trust change.

## Introduction

Healthcare organizations are beginning to pilot large language model (LLM) systems for guideline summarization, patient education, triage support, and evidence-linked question answering [1-3]. In real clinical communication, even small errors - misstated dosing ranges, missed contraindications, incorrect screening intervals, or shifted diagnostic thresholds - can create patient-safety risk when the output appears professionally sourced [4-6].

We use the term citation veneer to describe an answer that presents citation cues while containing incorrect or insufficiently supported content. This is not identical to general hallucination: it is a joint state in which the trust surface is misleading because source cues and content validity diverge.

Retrieval-augmented or citation-enabled generation can improve access to source material, but retrieval success and citation presence do not by themselves guarantee that the final answer is clinically correct, complete, numerically concordant, or materially supported by the cited passage. In clinical governance settings, this creates a monitoring problem: reviewers need compact audit artifacts that can identify when evidential cues remain present while substantive correctness degrades.

Conventional dashboards often report trust signals separately - accuracy in one panel, citation quality in another, and style or confidence markers elsewhere. Those summaries remain useful, but no existing lightweight monitoring view encodes correctness, evidential support, numeric concordance, style, and veneer jointly at the per-response level. Clinical informatics teams, educators, and quality-improvement groups, therefore, need a compact way to preserve several trust signals together for each answer during prompt tuning, retrieval-policy changes, and regression testing [2,5,7].

This gap is adjacent to retrieval-augmented generation and hallucination-reduction work. Retrieval-augmented generation was developed to tether language-model outputs to external knowledge [8], and benchmark studies show that retrieval-augmented generation systems still vary in faithfulness and answer quality [9]. Search-based revision approaches can reduce unsupported claims [10]. Fact-level and retrieval-augmented generation (RAG)-specific evaluators such as FActScore and RAGAS are valuable for measuring factual precision or retrieval-generation quality [11,12], but they do not by themselves provide a compact per-response clinical audit tag that preserves correctness, evidential support, numeric concordance, style, and citation veneer together.

Clinical AI reporting and governance guidance likewise emphasizes transparent reporting, intended use, validation, and monitoring for AI-enabled interventions [13-15]. The Clinical Evidence Audit Grid (CEAG) is designed to support that operational monitoring niche rather than to replace formal reporting guidelines, model benchmarks, or clinician judgment.

This study aims to develop and evaluate CEAG as a compact, analyst-facing audit view for detecting citation-present but incorrect outputs in guideline-grounded clinical and public-health LLM workflows. The primary objective is tool development and demonstration: to encode a response-level multi-signal audit state in a plain-text tag. Secondary aims are to examine whether CEAG makes citation veneer and local risk clusters visible in a synthetic stress-test benchmark, and to check whether the same tag logic remains usable in a small set of publicly sourced medical and public-health cases.

CEAG is intended for internal clinical informatics review, not for patient-facing display or autonomous clinical decision-making. Its role is to complement standard tables and charts by preserving the joint state of trust-relevant signals per response, helping reviewers localize risky clusters, compare response regimes, and separate stylistic changes from clinically meaningful trust changes [7].

## Materials and methods

Study design

We conducted a proof-of-concept methodological study of an audit representation, not a clinical trial, and not a live model-comparison benchmark. The main controlled benchmark used a synthetic, fictional guideline-style corpus containing 30 source passages and 120 grounded QA items. Each item had one gold answer and one evidence sentence, allowing exact control over correctness, citation support, and numeric concordance. No real patient data were used.

To assess whether the same audit logic could be applied to recognizable health-information materials, we also constructed a 12-case public check. Six cases were health-claim verification examples drawn from PubHealth-style items [16], and six were drawn from official public medical or public-health sources. The public cases were selected to include short claims, evidence-linked questions, and numeric or date-sensitive content; they were not intended to constitute a benchmark.

Model and generation settings

The controlled benchmark used scripted response regimes rather than outputs from a live LLM. This design was chosen to stress-test the audit surface under controlled combinations of correctness, citation cues, numeric drift, hedging, and ornamentation. Consequently, model name, API version, sampling temperature, seed, and decoding settings are not applicable to the controlled benchmark. We revised the manuscript to state this upfront because the earlier wording could be read as a live LLM evaluation.

Prompting/response regimes

Four response regimes were used to generate model-like output records. These are synthetic stress-test regimes, not claims about the behavior of a specific model. They were designed to emulate common prompts or presentation styles that clinical informatics teams may compare during monitoring.

The response-regime definitions used in the controlled benchmark are shown in Table 1. Because the benchmark did not query a live LLM, the entries below are prompt/generation paraphrases for scripted synthetic regimes rather than exact API prompts.

**Table 1:** Response-regime definitions. LLM: large language model

Regime	Definition	Prompt/generation paraphrase
RUSH	Fast-answer stress-test response regime	Answer directly with minimal checking; may preserve citation cues while allowing higher factual error. In this study, it is a scripted/synthetic regime, not a live LLM prompt.
VERIFY	Verification-focused stress-test response regime	Answer only after checking the evidence; lower error rate and fewer extra unsupported numbers. Scripted/synthetic regime.
POETIC	Stylistic-drift stress-test response regime	Use more figurative or hedged language; tests whether style changes fragment trust-state monitoring. Scripted/synthetic regime.
POSTER	Presentation-saturation stress-test response regime	Use poster-like emphasis and layout; tests whether ornamentation swamps substantive trust differences. Scripted/synthetic regime.

CEAG encoding and scoring

CEAG represents each response as a six-slot tag ordered CENHOV. Uppercase indicates that the signal is present (1), and lowercase indicates that the signal is absent (0). For example, CENhov indicates a clinically correct, evidence-supported response with grounded numbers and no hedging, ornamentation, or veneer. A tag such as cEnhoV marks CEAG-V: evidence appears supportive (E=1), but the content is incorrect (C=0).

The CEAG-V marker captures one specific subtype of misleading trust: supported-error citation veneer. Other risky states are also visible in the tag but are not counted as CEAG-V. For example, E=0 and C=0 denotes an unsupported error, and E=0 and C=1 denotes a correct but unsupported answer. We use CEAG-V narrowly to avoid implying that one marker captures all citation problems.

The CENHOV signal mapping and binary scoring rules are shown in Table 2.

**Table 2:** Signal mapping and scoring rules. CEAG: Clinical Evidence Audit Grid

Slot	Signal	Positive criterion	Borderline rule
C	correct	1 if the response matches the gold answer or guideline-grounded truth statement; 0 for wrong, materially incomplete, or misleading answers.	Partial answers that omit clinically required qualifiers are scored 0. CEAG flags presence of an error, not severity.
E	cite_ok	1 if the cited source materially supports the answer as stated; 0 for absent, irrelevant, or insufficient citation.	An answer can be correct with E=0 (unsupported truth) or wrong with E=0 (unsupported error).
N	num_grounded	1 if all numeric values required for the answer are present and concordant with evidence; 0 if any stated numeric value is wrong/unsupported or a clinically required number is omitted.	Omission of a required dose, threshold, age range, date, interval, or count is scored N=0.
H	hedge	1 if the response contains explicit uncertainty markers such as may, might, possibly, uncertain, or likely; otherwise 0.	Neutral caveats required by the source are not penalized unless they alter clinical meaning.
O	ornament	1 if the response uses poster-like, promotional, or rhetorically decorative styling that could inflate perceived confidence; otherwise 0.	Formatting alone is not clinical error; it is tracked to separate style from trust.
V	CEAG-V supported-error citation veneer	Derived as E=1 and C=0: the response is cited as if supported but is substantively incorrect.	This is one subtype of misleading trust. E=0/C=0 and E=0/C=1 are separately visible through the C and E slots but not counted as V.

Annotation process and reproducibility

Gold answers in the synthetic benchmark were created directly from the evidence sentences in the synthetic source passages. Gold answers in the 12-case public check were created from the public source pages listed in the dataset file. The full synthetic source-passage catalog, question catalog, public-case catalog, scoring guidelines, response-regime definitions, and scored output records are provided in the accompanying dataset files.

All C, E, N, H, O, and V labels in this proof-of-concept study were assigned by a single investigator using the rule-based criteria in Table 2; an independent multi-rater clinical annotation process was not performed, and inter-rater reliability was not calculated. This is a major limitation. The revised dataset package, therefore, makes the scoring rules explicit and marks all rows as single-investigator rule-based labels. Future validation should use at least two independent reviewers with clinical or public-health expertise, blind reviewers to the response regime where feasible, resolve disagreements by consensus or third-reviewer adjudication, and report inter-rater reliability, such as Cohen’s kappa for C, E, and N, before CEAG is used as a validated clinical QA instrument.

Borderline cases were handled as follows. A partially correct answer was scored C=0 if it omitted a clinically required qualifier. Numeric concordance was scored N=0 if any stated numeric value was wrong or unsupported, or if a clinically required dose, threshold, date, interval, count, or age range was omitted. Ornamental presentation was tracked separately from correctness because style may inflate perceived confidence without necessarily changing clinical content.

Grid ordering, Masked Delta, and statistical analysis

Two ordering rules were used. STRIPES_BY_DOC groups responses by source passage, making source-linked clusters visible. TIME_SERIES orders responses by the source-date metadata in the synthetic corpus. Ordering does not change any underlying rate; it changes only the reviewer's ability to spot local concentrations of joint states.

An adjacency match was defined as the percentage of horizontally or vertically adjacent grid cells that shared the same full CENHOV tag. It is a descriptive measure of local continuity, not a clinical endpoint. Masked Delta compares two regimes by applying an XOR operation to their CENHOV vectors and retaining only selected slots. In this manuscript, the content/trust mask retains C, E, N, and V; the style mask retains H and O.

Proportions are reported with 95% Wilson confidence intervals. The public case check is reported descriptively only because n=12 and one case changes the rate by 8.3 percentage points.

**Figure 1:**
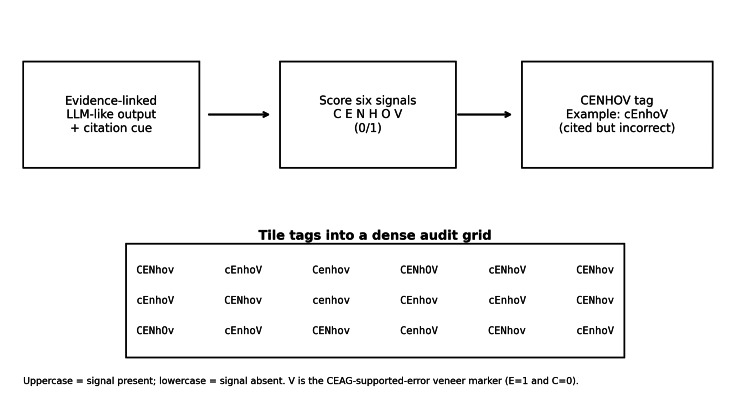
Clinical Evidence Audit Grid (CEAG) pipeline. One response is encoded as one fixed-length audit tag; a dataset becomes a compact grid that preserves joint audit state. LLM: large language model

The CEAG encoding and review workflow is shown in Figure 1.

## Results

The synthetic benchmark summary statistics, including Wilson 95% confidence intervals, are shown in Table 3.

**Table 3:** Public-case check summary with Wilson 95% confidence intervals (CIs).

Condition	n	Correct %	95% CI	Evidential support %	95% CI	Numeric concordance %	95% CI	CEAG-V %	95% CI
POETIC	12	83.3	55.2-95.3	100.0	75.7-100.0	41.7	19.3-68.0	16.7	4.7-44.8
POSTER	12	100.0	75.7-100.0	100.0	75.7-100.0	50.0	25.4-74.6	0.0	0.0-24.3
RUSH	12	33.3	13.8-60.9	100.0	75.7-100.0	25.0	8.9-53.2	66.7	39.1-86.2
VERIFY	12	100.0	75.7-100.0	100.0	75.7-100.0	50.0	25.4-74.6	0.0	0.0-24.3

The public-case check summary statistics, including Wilson 95% confidence intervals, are shown in Table 4.

**Table 4:** Synthetic benchmark summary with Wilson 95% confidence intervals (CIs).

Condition	n	Correct %	95% CI	Evidential support %	95% CI	Numeric concordance %	95% CI	CEAG-V %	95% CI
POETIC	120	81.7	73.8-87.6	75.0	66.6-81.9	20.8	14.5-28.9	15.0	9.7-22.5
POSTER	120	90.8	84.3-94.8	97.5	92.9-99.1	21.7	15.2-29.9	9.2	5.2-15.7
RUSH	120	73.3	64.8-80.4	93.3	87.4-96.6	20.8	14.5-28.9	25.8	18.8-34.3
VERIFY	120	96.7	91.7-98.7	100.0	96.9-100.0	31.7	24.0-40.4	3.3	1.3-8.3

The public check was small and should be interpreted as exploratory pilot data only. Given n=12, one case changes the rate by 8.3 percentage points, and the veneer estimates have wide uncertainty, approximately ±25 percentage points for some proportions. In those 12 cases, RUSH produced 66.7% (39.1-86.2) CEAG-V veneer. VERIFY and POSTER produced no CEAG-V cases in the same check, but their 95% upper bounds remain wide because of the small denominator. These results are suggestive of the same pattern observed in the synthetic stress test; they do not establish generalizability.

Ordering changed what became visible without changing any underlying rate. In the STRIPES_BY_DOC layout, the source block D4 - the synthetic medication reconciliation update after discharge - contained six CEAG-V-tagged RUSH responses among nine items (6/9), accounting for 19.4% of all RUSH veneer cases. This example does not prove that D4 is intrinsically risky; it shows that grouping related source items can reveal local concentrations that are erased by marginal bar charts.

**Figure 2:**
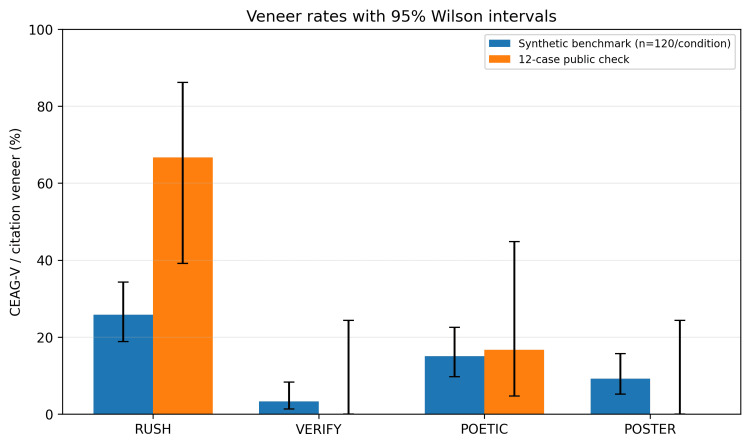
Veneer-oriented results on the synthetic benchmark and the 12-case public check. Error bars show 95% Wilson confidence intervals.

The veneer-oriented comparison between the synthetic benchmark and the 12-case public check is shown in Figure 2.

Masked Delta separated stylistic differences from substantive trust differences. A raw delta between VERIFY and POSTER was non-zero for every response because POSTER changed ornamentation almost everywhere. After masking style-related slots and retaining only C, E, N, and V, only 21.7% of responses differed between VERIFY and POSTER. By contrast, RUSH versus VERIFY showed 30.8% content/trust differences with only 10.8% style differences, a contrast that can help reviewers distinguish presentation shifts from trust-relevant changes.

**Figure 3:**
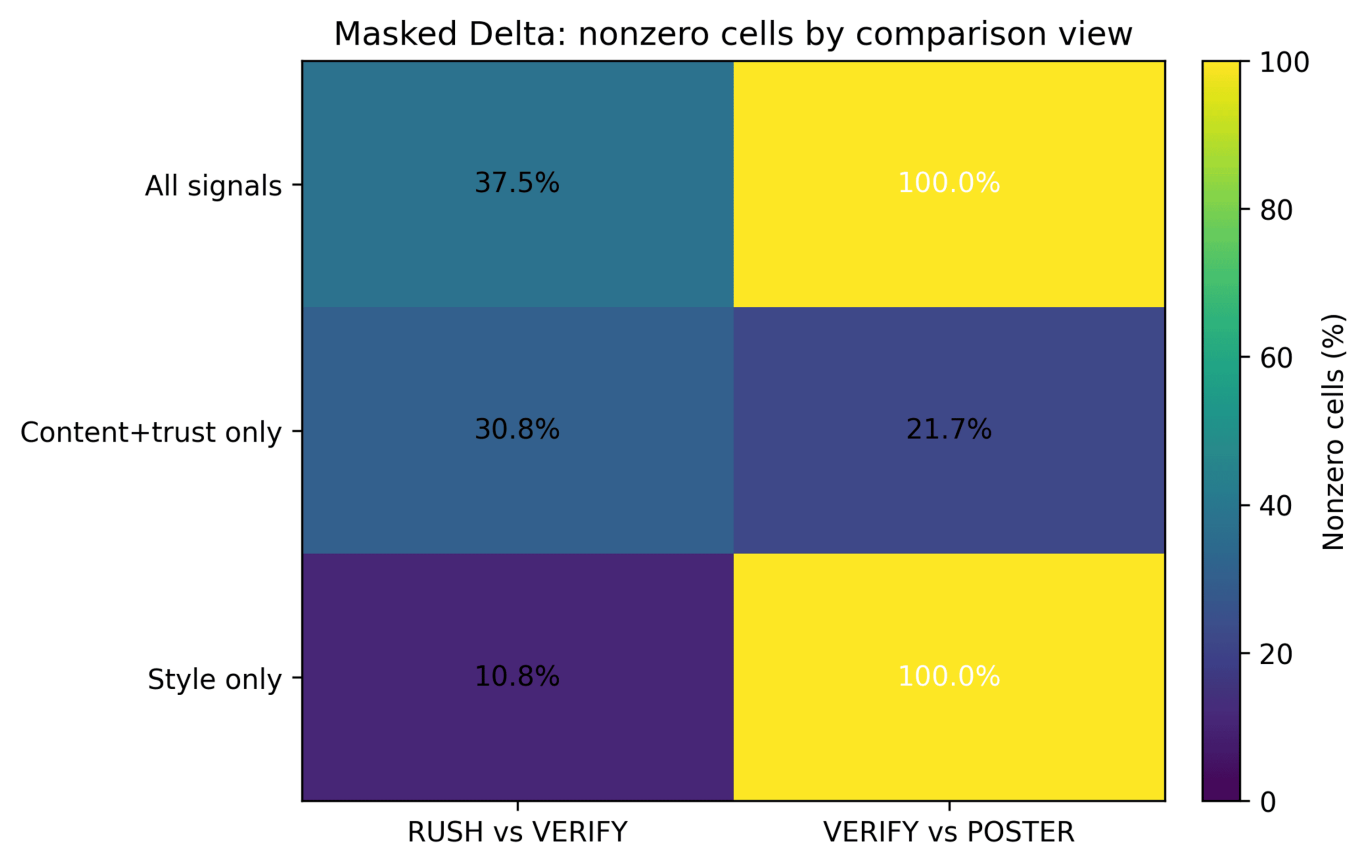
Masked Delta comparison. Nonzero cells report the percentage of responses whose retained slots differed between regimes.

The Masked Delta comparison of content/trust differences and style differences is shown in Figure 3.

## Discussion

CEAG is best understood as a monitoring surface for guideline-grounded clinical and public-health LLM workflows. It does not validate a model, establish clinical safety, or replace expert review. Rather, it offers a compact way to preserve the joint response state that often matters in quality assurance: correctness, evidence support, numeric concordance, style, and the specific supported-error veneer state.

CEAG complements rather than replaces existing evaluation methods. FActScore decomposes generated text into atomic facts and estimates the proportion supported by external sources [12]. RAGAS evaluates retrieval-augmented generation pipelines with reference-free metrics for retrieval and generation quality [13]. CEAG is less granular than these frameworks, but it preserves a per-response joint state in a tag that can be scanned, copied into issue trackers, and compared across response regimes. The trade-off is intentional: CEAG is designed as an audit surface for triage and review, not as a scalar faithfulness score.

Table 5 summarizes the representational affordances of CEAG relative to grouped bars and separated heatmaps. This is a comparative analysis of audit affordances, not a usability experiment. Grouped bars remain the clearest way to communicate headline rates, and separated heatmaps remain useful when one metric is already known to be central. CEAG is useful when the audit target is a conjunction of signals, such as C=0, E=1, and V=1.

The comparative audit affordances of grouped bars, separated heatmaps, and CEAG/Masked Delta are summarized in Table 5.

**Table 5:** Comparative analysis of audit affordances. CEAG: Clinical Evidence Audit Grid

Audit property	Grouped bars	Separated heatmaps	CEAG/Masked Delta
Marginal per-condition rates	Direct	Indirect	Indirect
Per-response joint state	Absent	Fragmented across panels	Direct
Explicit CEAG-V mark	Absent	Requires cross-panel conjunction	Direct (V slot)
Localization of clusters	Absent	Partial	Direct via ordering
Style-vs-trust comparison	Absent	Weak	Direct via Masked Delta

Potential healthcare uses should be framed as hypotheses for future validation rather than as demonstrated deployment readiness. In a hospital quality-assurance team, clinical informatics group, public-health communication team, or medical education program, CEAG could support pre-deployment review of guideline summaries, regression testing after prompt or retrieval updates, and post-deployment monitoring when citation-attribution remains high while clinical correctness degrades. This use case is consistent with clinical AI reporting and governance guidance that asks developers to specify intended use, validation scope, monitoring plans, and limitations for AI-enabled clinical systems [13-15].

Accessibility is also part of the clinical workflow. Because CENHOV is a plain-text tag, it can be exported as an HTML table, spreadsheet, issue-tracker field, or CSV rather than only as a dense visual grid. Production implementations should include text-equivalent tables, keyboard navigation, accessible summaries of clusters, and screen-reader-friendly descriptions of each cell's C, E, N, H, O, and V states [17,18].

Limitations

First, binary encoding may understate clinically meaningful severity. CEAG flags the presence of an error state, not the magnitude of potential harm. Clinical risk stratification remains a downstream task rather than an output of the current CEAG tag. A minor screening-interval discrepancy and a life-threatening dosing error may both receive C=0 or V=1, even though their clinical implications differ. Future work should test ordinal or severity-weighted extensions, such as C=0/1/2 for wrong/partial/correct or an additional severity field.

Second, scoring validity remains limited. All labels in this proof-of-concept were assigned by a single investigator using rule-based criteria. We did not perform independent clinical annotation and did not calculate inter-rater reliability. Before CEAG can be treated as a validated clinical QA method, future work should use two or more independent clinical reviewers, report agreement for C, E, and N, and resolve disagreements by consensus.

Third, the controlled benchmark is synthetic, and the public-case check is small. The synthetic corpus is useful for exact stress testing but does not capture the ambiguity of real clinical communication. The 12-case public check is descriptive and has wide uncertainty; it should not be interpreted as proof of generalizability.

Fourth, this study does not evaluate a specific live LLM model. Prompt sensitivity, stochastic variation across runs, provider-side model updates, retrieval-system behavior, and model versioning may all affect reproducibility in real deployments. The present data demonstrate an audit representation and a proof-of-concept scoring workflow, not model-specific performance.

Fifth, we provide a task-oriented analytical comparison rather than a formal user study. A next study should measure detection time, miss rate, reviewer confidence, and correction workload in actual clinical QA or public-health communication review workflows.

## Conclusions

CEAG turns multi-signal auditing of guideline-grounded clinical and public-health LLM-like outputs into a compact analyst-facing view. Its practical value lies in preserving the joint state of trust-relevant signals so that supported-error citation veneer, grounded-looking hallucinations, and localized clusters of risk can be identified for review.

The next concrete step is a prospective reviewer study with hospital QA teams, clinical informatics groups, medical educators, or public-health communication teams. Such a study should compare CEAG against grouped bars, separated heatmaps, and ordinary tables for citation-veneer triage tasks and should include independent clinical raters to quantify scoring reliability.
